# Decreased Level of Blood MicroRNA-133b in Men with Opioid Use Disorder on Methadone Maintenance Therapy

**DOI:** 10.3390/jcm8081105

**Published:** 2019-07-25

**Authors:** Chih-Wei Hsu, Tiao-Lai Huang, Meng-Chang Tsai

**Affiliations:** Department of Psychiatry, Kaohsiung Chang Gung Memorial Hospital and Chang Gung University College of Medicine, Kaohsiung 833, Taiwan

**Keywords:** addiction, etiology, heroin, MicroRNA, methadone maintenance therapy, opioid use disorder

## Abstract

Although previous animal studies have indicated that certain micro ribonucleic acids (microRNAs) play a part in the pathway of opioid addiction, whether such findings extend to human models is yet unknown. This study aims to investigate the important microRNA expressions in patients with opioid use disorder (OUD) on methadone maintenance treatment (MMT) compared to healthy controls and analyze the correlation between microRNAs and opioid characteristics among the patients. We recruited 50 patients and 25 controls, and both groups were matched regarding gender, age, and body mass index. Serum microRNAs (miR-133b, miR-23b, miR-190, miR-206, miR-210, and miR-21) were measured. The age of OUD onset, duration of MMT participation, and recent daily methadone dosage were considered the opioid characteristics. We adopted the *t*-test to compare the difference between patients and controls and Pearson’s correlation to evaluate the association between microRNAs and opioid profiles. Only the level of miR-133b in OUD patients on MMT was significantly lower than that in healthy controls. We did not detect differences of any other microRNA expressions between the two groups. Furthermore, we found no evidence to support the association between microRNAs and opioid characteristics. This study indicates that miR-133b values may be decreased in OUD patients on MMT.

## 1. Introduction

Opioid use disorder (OUD) is a major public health issue and has recently been estimated to afflict 0.37% of the adult population [1]. Heroin users with OUD have distinctly associated morbidity and mortality rates [2], such as a high risk for infection of human immunodeficiency virus (HIV) or hepatitis via intravenous injection [3], and a four- to eight-fold risk of all-cause mortality compared to the general population [4,5]. Typical management of OUD involves methadone maintenance treatment (MMT) [6], which can block the euphoric effects and suppress the withdrawal symptoms associated with heroin [7]. However, using MMT for heroin addictions is still controversial [8] and carries a risk of relapse for heroin use. Therefore, further investigation into the biomarkers and mechanisms of opioid addiction in patients with OUD is vital.

Micro-ribonucleic acid (microRNA or miR) is a class of non-coding small RNAs that acts as a regulator of target gene products via messenger RNA (mRNA) cleavage or translational repression [9]. MicroRNA is remarkably stable in the blood and even under multiple freeze–thaw cycles or extended storage, which exhibits advantageous characteristics as a potential biomarker for clinical use [9]. Moreover, recent studies have reported that certain microRNAs (such as miR-133b, miR-23b, miR-190, miR-206, miR-210, and miR-21) can regulate opioid receptors through methylation and chromatin remodeling [10,11] and impact the dopamine system [12], synaptic plasticity, and neuronal cell development [13,14], as well as neuroimmune system interaction [15]. The modulation of opioid receptors can then finally be connected to the drug addiction process [16], and therefore, understanding the expression of blood miRNAs may facilitate the development of novel diagnostic tools [9]. However, most previous studies focusing on the pathway of opioid dependence have been animal studies and have had limited human data; for example, opioid decreased the expression of miR-133b in zebrafish embryos [17]. Therefore, we do not understand whether those microRNA expressions found in animal designs can be extended to human models. If such findings could be applied to humans, we further explored whether the microRNAs are associated with the characteristics of OUD patients.

To clarify certain microRNA levels in humans and their relationships with opioid use, we designed this study to investigate the serum levels of microRNAs (miR-133b, miR-23b, miR-190, miR-206, miR-210, and miR-21) in OUD patients undergoing MMT compared to healthy controls and then analyzed the association between microRNA expressions and opioid use among the participating patients.

## 2. Materials and Methods

### 2.1. Patients and Controls

The study was conducted in accordance with the Declaration of Helsinki, and the protocol was approved by the Institutional Review Board of Chang Gung Memorial Hospital (Project identification code 201509344A3). We recruited 50 outpatients diagnosed with OUD that were receiving MMT through the Structured Clinical Interview of the *Diagnostic and Statistical Manual of Mental Disorders, Fifth Edition* (DSM-5) and 25 healthy controls enlisted from November 2016 to October 2017 from Kaohsiung Chang Gung Memorial Hospital Medical Center in Taiwan. All participants agreed to the study protocol approved that was previously by the hospital ethics committee and then provided their informed written consent. We reviewed their background records (onset age of OUD and duration of MMT), current methadone dosage, and routine physical examinations, including gender, age, weight, height, and body mass index (BMI), at the time of enrolment. We excluded any individuals with a psychiatric history (such as schizophrenia, bipolar disorder, major depressive disorder, anxiety disorder, or substance use disorder beside heroin or tobacco use disorder) or physical comorbidity (such as human immunodeficiency virus or chronic pain problem treated with non-steroidal anti-inflammatory drugs or opioid analgesics).

### 2.2. Laboratory Data

The levels of miR-133b, miR-23b, miR-190, miR-206, miR-210, and miR-21 in the blood were the main outcome of our study. We collected venous blood samples from all participants in a fasting state (for at least 8 h), and they were demonstrated to not significantly affect the overall serum miRNA profiles [18]. We separated red blood cells (RBC) from the whole blood with the lysing process using a RBC Lysis buffer (SG5011000, RBC Bioscience Corp, Taipei, TW) and centrifugation at 3000 revolutions per minute for 1 min. Then miRNA was isolated and purified with the *mir*Vana^TM^ miRNA Isolation Kit (Ambion, Life Technologies, Carlsbad, CA, USA). Finally, we utilized TaqMan^®^ Advanced miRNA cDNA Synthesis Kit (A28007, Applied Biosystems, Foster City, CA, USA) to prepare the complementary deoxyribonucleic acid (cDNA) from mature microRNAs, which included the following four steps: poly A tailing reaction, ligation, reverse transcription, and miR-amplification on a Veriti^®^ thermal cycler (Applied Biosystems).

We detected miRNA using TaqMan^®^ Advanced miRNA Assays (A25576, Applied Biosystems) and quantified miR-133b, miR-23b, miR-190, miR-206, miR-210, and miR-21 expression levels through quantitative real-time polymerase chain reaction (qPCR) analysis with TaqMan^®^ Fast Advanced Master Mix (4444557, Applied Biosystems) on a 7500 Fast Real-Time PCR System (Applied Biosystems). We normalized total RNA input based on the Ct values obtained for cel-miR-39 (10 pM per RNA sample), an exogenous control used to monitor extraction efficiency or sample input amount in this analysis. We calculated relative expression levels of microRNAs by 2^^−ΔΔCt^. We performed all processes and analyses according to the respective manufacturers’ instructions in the same laboratory.

### 2.3. Statistical Analysis

All data were analyzed using MedCalc Statistical Software version 18.5 (MedCalc Software bvba, Ostend, Belgium; http://www.medcalc.org; 2018). Continuous variables were expressed as mean ± standard deviation; independent samples t-test was used to compare data between patients and control subjects; and Pearson’s correlation coefficient was adopted to evaluate the relationship between the profile of OUD patients on MMT and microRNA levels. A two-tailed *p*-value < 0.05 was considered statistically significant. For multiple testing between the two groups in the study, a false discovery rate (FDR) was employed to adjust the p-value while it was significant in the unadjusted comparison [19].

## 3. Results

All of the 50 patients with OUD on MMT and the 25 healthy controls in this study were males, and no significant differences were found between the mean age (44.76 ± 6.18 vs. 46.32 ± 6.16) and BMI (24.92 ± 3.55 vs. 24.65 ± 3.23) of the two groups. The onset age of the OUD patients was 25.54 ± 6.03 years old, and their duration of MMT was 3.96 ± 3.33 years with a methadone dosage of 65.00 ± 26.28 mg per day in the most recent month before the blood tests. The mean level of miR-133b in OUD patients was significantly lower than that in healthy controls (0.78 ± 0.87 vs. 1.20 ± 0.75, *p* = 0.041), but this difference was no longer significant after adjusting with FDR (*p* = 0.328). We did not observe any difference in microRNA expressions related to other blood levels, including miR-23b (0.97 ± 1.89 vs. 1.72 ± 2.28), miR-190 (1.33 ± 1.45 vs. 1.40 ± 1.19), miR-206 (2.57 ± 9.68 vs. 1.29 ± 0.95), miR-210 (1.32 ± 0.85 vs. 1.26 ± 0.92), and miR-21 (1.05 ± 0.48 vs. 1.07 ± 0.36) (Table 1). Table 2 demonstrates the association between the profiles of included patients (onset age of OUD, duration of MMT, and methadone dosage in the past 1 month) and microRNA levels (miR-133b, miR-23b, miR-190, miR-206, miR-210, and miR-21). We did not observe any associations between opioid profiles and microRNA levels in patients with OUD. Furthermore, no participants reported an adverse event or dropped out while blood was collected, or history was taken in this study.

## 4. Discussion

In this study, we investigated the microRNA levels between OUD patients receiving MMT and healthy controls and analyzed the association between those patients′ profiles and their microRNA expressions. To the best of our knowledge, this article is the first to explore these microRNA differences between OUD patients and controls. Our results indicated that only the miR-133b level may be lower in patients with OUD. We also did not detect any association between those profiles and microRNA expressions.

In the past, several animal studies have reported that miR-133b may play an important role in the addiction process due to the interaction between the dopamine system and the use of morphine [12,20,21]. First, miR-133b downregulates the homeodomain transcription factor Pitx3 (a target in the dopaminergic system), which regulates the differentiation and maturation of dopaminergic neurons by activating the expression of tyrosine hydroxylase (TH) and the dopamine transporter (DAT) [22,23]. Second, miR-133b levels of zebrafish μ-opioid receptor (zfMOR) in the embryos were observed to be lower after injecting morphine [21]. Altogether, morphine treatment may decrease miR-133b, then increase Pitx3 and active TH and DAT, which induces a dopaminergic-related drug addiction. Although this mechanism hypothesis comes from animal studies, our human findings can partially support these previous results and provide a potential link between animal hypotheses and the clinical phenomena of OUD patients on MMT, which is lower miR-133b levels. Moreover, a previous study indicated that a lower miR-133b level can serve as a biomarker of Parkinson’s disease [24]. Our result may mean that the lower blood level of miR-133b will be a potential candidate biomarker to diagnose OUD in clinical practice.

A literary review also demonstrated that opioid addiction was associated with miR-23b and miR-190, which affected the expression of opioid receptors and altered the neuronal activity. More detailed, long-term morphine treatment increased miR-23b expression and suppressed the association of mu-opioid receptor messenger RNA by binding the sites on the 3’-untranslated region [25,26]. Furthermore, fentanyl and morphine decreased miR-190 expression, induced extracellular signal-regulated kinase (ERK) phosphorylation, and impaired NeuroD activity [27,28]. Finally, the activity of NeuroD contributed to the stability of dendritic spines in addiction [29]. However, our results showed a marginally low level of miR-23b in OUD patients but no difference of miR-190 between patients and healthy controls, which indicates that the miR-23b (directly regulate the mu-opioid receptor) may play a more critical role than miR-190 (affect neuronal activity through NeuroD) in patients with opioid addiction. Besides, limited samples in our trial may have reduced the significance of our findings.

In this study, we investigated two microRNAs, miR-206 and miR-210, to determine whether they had a correlation with addiction in OUD patients via brain-derived neurotrophic factor (BDNF). BDNF is known to play a key role in the motivational effects of addictive medications [30], and a previous study demonstrated a relationship between BDNF and addictive behavior in patients with OUD [31]. With regard to miR-206, several previous studies indicated a negative expression of BDNF via directly suppressing the mRNA binding sites [32]. Furthermore, Fasanaro et al. have identified BDNF as a potential target of miR-210 responding to hypoxia through modulating cell survival [33]. In this study, lack of evidence favors a significant association between these BDNF-related microRNAs and addiction in patients with OUD, but the miR-206 seems to be marginally higher in OUD patients. The phenomenon may hint that miR-206 plays an important role because of regulating BDNF directly. We also looked at miR-21 for its role in morphine-induced inflammation [34]. However, the current results of our study indicated no difference between OUD patients and controls, which did not support previous findings. It may be due to the different mechanism. The miR-21 expression is associated with pain-related morphine use in the prior study but not addiction-related methadone use in this study.

We analyzed the association between microRNAs expression and opioid profiles among OUD patients, which included onset age, duration of MMT, and recent methadone dosage in this study. Various evidence indicated that those profiles may be features of OUD severity. For example, opioid-dependent men with an early onset age had a greater severity of opioid dependence [35]; meanwhile, significantly reduced brain activity to heroin-related minus neural cues in the bilateral cortical caudate with long-term MMT and higher total methadone consumption improved heroin-craving response [36]. In this study, we hypothesized that those opioid profiles could affect addiction, and microRNAs may play a role in the phenomenon. However, we were unable to find the association to prove it. Only methadone dosage and miR-133b had a marginal significance regarding negative correlation (−0.27 with −0.51 to 0.01 95% confidence interval). We suppose that the phenomenon plus previous paragraphs may hint at the dopamine system having a more important role among addiction than other neuro-biological pathways.

This study had several limitations. First, all patients in our study were males, so the same findings may not be extended to women. In the future, we will need more samples from female groups to confirm our findings. Second, although we investigated the association between general profiles (onset age, duration of MMT, and methadone dosage in the past month) and microRNAs to examine the effect of addictive severity to microRNA results, another important confounding factor, that is, prior heroin dosage before MMT, was lacking. This lack of data was because the dose reported from those patients was difficult to measure (various forms of heroin, such as injection or inhalation) and may hinder our severity assessment of OUD patients. Furthermore, we were unable to detect that the differences of microRNAs expressions is the result of OUD alone, MMT use, or an interaction of OUD and MMT. Further studies are required to compare the microRNAs expression between OUD only, OUD with MMT, and healthy controls. Third, we excluded OUD patients with other major psychiatric disorder but did not screen personality disorders among them. OUD patients comorbid with personality disorders, such as borderline personality disorder, may confound our results. Fourth, the samples in previous studies mainly came from the peripheral blood [9,37], but measuring microRNA levels from different locations, such as brain tissue and peripheral blood (RBC, plasma, or serum), may result in some discrepancies. Whether peripheral microRNA levels in the blood can directly reflect the levels in the brain is also unclear. Fifth, sample sizes were too small, which may impede adequate statistical analysis to detect differences in microRNA levels between the two groups. Finally, none of the 75 enrolled participants dropped out of this study, which may be attributed to the short period of the research program (less than half a day) and the simple approach adopted (one-time examination, including blood collection and history taking).

## 5. Conclusions

In conclusion, this study provides evidence that miR-133b levels may be lower in OUD patients on MMT. In the future, additional human studies with more samples are needed to confirm our findings and explore the detailed mechanism underlying the association between miR-133b and opioid addiction in patients with OUD.

## Figures and Tables

**Table 1:** Characteristics and clinical data of patients with opioid use disorder and healthy controls in the study.

	OUD (*N* = 50, male)	Controls (*N* = 25, male)	*p*	Adjusted *p* ^a^
Age (years)	44.76 ± 6.18	46.32 ± 6.16	0.305	0.720
BMI (kg/m^2^)	24.92 ± 3.55	24.65 ± 3.23	0.748	0.866
Onset age (years)	25.54 ± 6.03	NA		
Duration of MMT (years)	3.96 ± 3.33	NA		
Methadone dosage in the past one month (mg/day)	65.00 ± 26.28	NA		
miR-133b	0.78 ± 0.87	1.20 ± 0.75	0.041 *	0.328
miR-23b	0.97 ± 1.89	1.72 ± 2.28	0.133	0.532
miR-190	1.33 ± 1.45	1.40 ± 1.19	0.829	0.866
miR-206	2.57 ± 9.68	1.29 ± 0.95	0.360	0.720
miR-210	1.32 ± 0.85	1.26 ± 0.92	0.792	0.866
miR-21	1.05 ± 0.48	1.07 ± 0.36	0.866	0.866

Data values are given as mean ± standard deviation; * *p* < 0.05; ^a^ adjusted p value using method of false discovery rate; Abbreviation: BMI: body mass index, miR: micro ribonucleic acid, MMT: methadone maintenance therapy, NA: not applicable, OUD: opioid use disorder.

**Table 2:** Correlations between microRNA levels and opioid profiles of patients with opioid use disorder.

	Onset Age	*p*	Duration of MMT	*p*	Methadone Dosage	*p*
miR-133b	0.17 (−0.12, 0.42)	0.249	−0.18 (−0.44, 0.10)	0.201	−0.27 (−0.51, 0.01)	0.055
miR-23b	−0.16 (−0.42, 0.12)	0.256	0.17 (−0.11, 0.43)	0.240	−0.08 (−0.35, 0.21)	0.594
miR-190	0.25 (−0.03, 0.50)	0.074	0.02 (−0.26, 0.30)	0.897	−0.01 (−0.29, 0.27)	0.955
miR-206	−0.11 (−0.38, 0.17)	0.431	−0.10 (−0.36, 0.19)	0.511	−0.17 (−0.42, 0.12)	0.249
miR-210	−0.14 (−0.41, 0.14)	0.322	−0.26 (−0.50, 0.02)	0.065	0.04 (−0.24, 0.31)	0.793
miR-21	0.03 (−0.25, 0.31)	0.818	−0.18 (−0.44, 0.10)	0.213	−0.18 (−0.44, 0.11)	0.216

Data values are given as correlation coefficient with 95% confidence interval; Abbreviation: miR: micro ribonucleic acid, MMT: methadone maintenance therapy.
